# Dietary Curcumin: Correlation between Bioavailability and Health Potential

**DOI:** 10.3390/nu11092147

**Published:** 2019-09-08

**Authors:** Michele Dei Cas, Riccardo Ghidoni

**Affiliations:** Department of Health Sciences, Università degli Studi di Milano, 20142 Milan, Italy

**Keywords:** curcumin, pharmacokinetic, bioavailability, nutrient, nutraceutical

## Abstract

The yellow pigment curcumin, extracted from turmeric, is a renowned polyphenol with a broad spectrum of health properties such as antioxidant, anti-inflammatory, anti-cancer, antidiabetic, hepatoprotective, anti-allergic, anti-dermatophyte, and neuroprotective. However, these properties are followed by a poor pharmacokinetic profile which compromises its therapeutic potential. The association of low absorption by the small intestine and the extensive reductive and conjugative metabolism in the liver dramatically weakens the oral bioavailability. Several strategies such as inhibition of curcumin metabolism with adjuvants as well as novel solid and liquid oral delivery systems have been tried to counteract curcumin poor absorption and rapid elimination from the body. Some of these drug deliveries can successfully enhance the solubility, extending the residence in plasma, improving the pharmacokinetic profile and the cellular uptake.

## 1. Introduction

**Figure 1:**
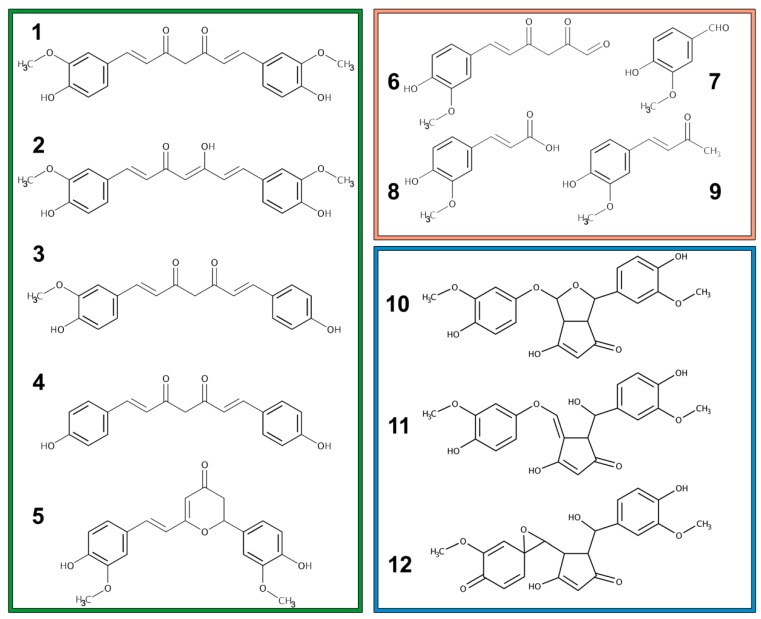
Chemical structures of curcuminoids (green box): (1) Curcumin keto-form, (2) Curcumin enolic-form, (3) demethoxycurcumin, (4) bis-demethoxycurcumin, and (5) cyclo-curcumin. Chemical structures of curcumin degradation products (orange box): (6) trans-6-(4’-hydroxy-3’-methoxyphenyl)-2,4-dioxo-5-hexenal, (7) vanillin, (8) ferulic acid, and (9) feruloyl-methane. Chemical structures of some curcumin autoxidation products (blue box): (10) bicyclopentadione, (11) vinylether, and (12) spiroepoxide.

Curcumin is a yellowish pigment and a well-known polyphenol found in the rhizome of turmeric and other *Curcuma* spp. Curcumin is usually present in the plant of the Zingiberaceae family with the related compounds demethoxycurcumin, bis-demethoxycurcumin, and cyclo-curcumin (Figure 1, green box). Together, these four compounds are referred to as curcuminoids [1]. Noticeable, for curcuminoid content in the roots, are the species *Curcuma zedoaria* (>100 μg/g), *Curcuma longa* (1–2 μg/g) and *Curcuma aromatica* (0.1 μg/g) [2].

Curcumin, chemically known as [1,7-bis(4-hydroxy-3-methoxyphenyl)-1,6-heptadiene-3,5-dione] or diferuloylmethane, exhibits keto-enol tautomerism dependent on the solution pH: at pH < 7 the keto-form is the principal, while at pH > 7 it occurs in the enol form [3].

Curcumin is reported to have three different pKa values: the first (pKa 7.7–8.5) and second (pKa 8.5–10.4) values are from the two phenolic hydroxy groups, and the third (pKa 9.5–10.7) value is from the enolic proton [1]. It is chemically stable between pH 1–6, but it is practically insoluble in water in this pH range. Autoxidation reaction, occurring a physiological pH, lead to a series of bicyclopentadione products (Figure 1, blue box) in which the 7-carbon chain has undergone oxygenation and double cyclization. Early autoxidative degradation products mediate the activity on topoisomerase, target of many chemotherapy agent already present in clinical practice [4]. Another minor pathway of cleavage which take place in alkaline media with the degradation into trans-6-(4’-hydroxy-3’-methoxyphenyl)-2,4-dioxo-5-hexenal and secondly into vanillin, ferulic acid, and feruloyl-methane (Figure 1, orange box). Stability is indeed related to the undissociated form of the hydroxy groups [2]. Oxidative degradation and modification can also be induced by light absorption (photo-degradation). Degradation reactions dramatically change curcumin structure and properties affecting its pharmacokinetic and pharmacodynamic behavior [5].

In *Curcuma longa*, crude extract curcuminoid makes up 1–6% of turmeric by weight, distributed in 60–70% curcumin, 20–27% demethoxycurcumin, and 10–15% bis-demethoxycurcumin [6] whereas commercially available curcumin contains about 77% in curcuminoids [2].

Turmeric has been traditionally used as a dye, a spice, and as an anti-inflammatory remedy in both Indian and Chinese medicine. The variety of health properties such as antioxidant, anti-inflammation, anti-cancer, antidiabetic, hepatoprotective, anti-allergic, anti-dermatophyte, and neuroprotective effects have been attributed to curcumin, the active principle of turmeric [7,8]. From a molecular point of view, curcumin may mediate its pharmacological activities through Janus kinase/signal transducers and activators of transcription (JAK-STAT), nuclear factor kappa B (NF-kB), protein kinase B (AKT or PKB), transforming growth factor b (TGF-b), and mammalian target of rapamycin (mTOR) [7,9,10,11,12,13]. In particular, the transcription factor Nrf2 and NF-kB regulate: (1) the inhibition of the transcription factor NF-kB mediates anti-inflammation and (2) the induction of Nrf2 signalling pathways promotes antioxidant defence mechanisms and production of phase II enzymes [2]. Anticarcinogenic effects of curcumin are also related to an increase in the p53 levels and thus in pro-apoptotic Bax and cytochrome C. The suppression of proliferation and a cell-cycle arrest can be modulated by curcumin also through p53 independent pathway such as the inhibition of NFkB inhibitor a (IkBa), B-cell lymphoma 2 (Bcl-2), B-cell lymphoma-extra large (Bcl-xl), cyclin D1, and interleukin 6 (IL6). Moreover, apoptosis can be initiated by curcumin by the increased cleavage of poly(ADP-ribose) polymerase (PARP) [2].

## 2. Poor Pharmacokinetic Profile of Curcumin after Oral Intake

It is well accepted that curcumin itself displays poor solubility in water, chemical instability, and a lowly pharmacokinetic profile. Despite its efficacy and safety, the therapeutic potential of curcumin is indeed still debated due to a relatively poor bioavailability in humans, even when administered at high dosage (12 g/day) [14]. Generally, the oral bioavailability of curcumin is low due to a relatively low absorption by small intestine coupled to an extensive reductive and conjugative metabolism in the liver and an elimination through the gall bladder. The poor bioavailability is also exacerbated by the curcumin bindings to enterocyte proteins that can modify its structure [5].

The bioavailability of nutraceuticals is a function strictly dependent on transformation in the gastrointestinal tract and bioaccessibility. The transformation refers to the amount of compound that remains in a bioactive form in the intestinal phase, whereas bioaccessibility is the amount of active compound accessible for absorption. Bioavailability, indeed, is the product of these two factors [15].

### 2.1. Metabolism

**Figure 2:**
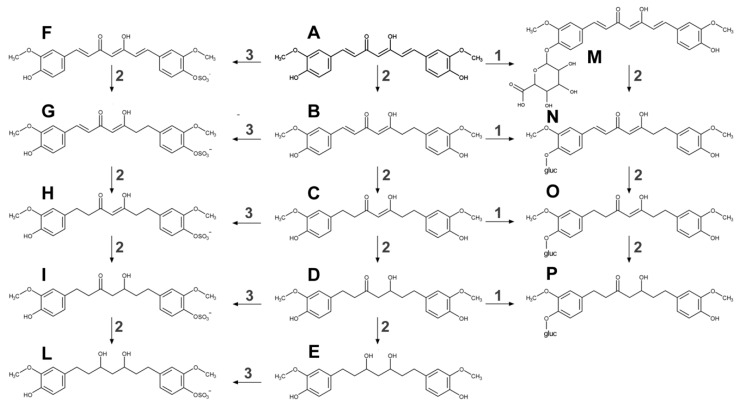
Curcumin phase I and II metabolism in a living organism. Curcumin (**A**) undergoes several reactions of reduction by a reductase (2) to dihydrocurcumin (**B**), tetrahydrocurcumin (**C**), hexahydrocurcumin (**D**) and octahydrocurcumin (**E**). Curcumin can be both conjugating to any of the hydroxyl groups, with glucuronic acid by glucuronosyltransferase (1) or sulfate by sulfotransferase (3). Phase II products are the followings: curcumin glucuronide (**M**), dihydrocurcumin glucuronide (**N**), tetrahydrocurcumin glucuronide (**O**), hexahydrocurcumin glucuronide (**P**), curcumin sulfate (**F**), dihydrocurcumin sulfate (**G**), tetrahydrocurcumin sulfate (**H**), hexahydrocurcumin sulfate (**I**), and octahydrocurcumin sulfate (**L**). In structure N, O, P ‘gluc’ is referred to as glucuronic acid.

Curcumin undergoes extensive phase I and II biotransformation (Figure 2). The liver is indicated as the primary site of metabolism for curcumin, together with intestine and gut microbiota [16]. Curcumin’s double bonds are subsequently reduced in enterocytes and hepatocytes by a reductase to dihydrocurcumin, tetrahydrocurcumin, hexa-hydrocurcumin, and octahydrocurcumin.

Moreover, secondary biliary metabolism was dihydro-ferulic acid and ferulic acid [2,14]. Phase II metabolism is quite active, in the intestinal and hepatic cytosol, on both curcumin and its phase I metabolites especially by conjugation with glucuronic acid and sulfate at the phenolic site. Curcumin is sulfated by SULTs in the cytosol, mainly SULT1A1 and SULT1A3, whereas UGTs catalyse the glucuronidation of curcumin in the intestinal and hepatic microsomes. Dihydrocurcumin, tetrahydrocurcumin, and hexa-hydrocurcumin exist in both free-forms or as glucuronides [2,6,14,16].

Curcumin also undergoes an alternative metabolism by intestinal microbiota such as *Escherichia coli* and *Blautia* sp. *Escherichia coli* was found to be active by an NADPH dependent reductase in a two-step reduction pathway from curcumin to dihydrocurcumin then to tetrahydrocurcumin [17]. *Blautia* sp. produces curcumin demethylation into two derivatives: demethylcurcumin and bis-demethylcurcumin [18]. Interestingly, several studies report that the poly-pharmacology of curcumin can be attributed to its metabolites, which are recognized as anti-oxidant, anti-inflammatory, antitumor, cardioprotective, and anti-diabetic [5,19,20,21,22,23,24].

### 2.2. Absorption, Bioavailability, and Tissue Concentration

Several studies have been performed using curcumin oral supplement to investigate its absorption in both human and laboratory animals.

Curcumin was prescribed orally for three months in 25 patients with high-risk or pre-malignant lesions. Patients enrolled for this study presented one of the following pathologies: resected urinary bladder cancer, arsenic Bowen’s disease of the skin, uterine cervical intraepithelial neoplasm, oral leucoplakia, and intestinal metaplasia of the stomach. Curcumin’s serum peak was found at 1 to 2 h after oral intake. Then gradually declined within 12 h. The average serum concentrations after taking 4, 6, and 8 g of curcumin were 0.51 ± 0.11 μM, 0.63 ± 0.06 μM, and 1.77 ± 1.87 μM, respectively. Excretion of curcumin in urine was inappreciable [25].

Healthy volunteers (*n* = 24) were administered with a single dose of curcumin standardized powder extract with doses ranging from 0.5 to 12 g. The dose was followed by a cup of water and a standard meal containing dietary fat. No curcumin was detected in the serum of participants administered with a dose inferior to 8 g. In 2/24 subjects, curcumin was found at a level of about 30 (1 h), 40 (2 h), and 50 (4 h) ng/mL after a dose of 10 g whereas level of about 30 (1 h), 60 (2 h), and 50 (4 h) ng/mL after a dose of 12 g [26].

Patients (*n* = 15) with chemotherapy-refractory colorectal cancer received Curcuma extract daily for up to 4 months at doses between 0.4 and 2.2 g/day, containing 36–180 mg of pure curcumin. Curcumin was recovered only in faeces after 29 days from consumption with a concentration at about 64 and 1054 nmol/g depending on dosage (1.7 to 2.2 g of extract or 144 to 180 mg of curcumin). In both blood or urine curcumin or its metabolites were not detectable [27].

Another study on chemotherapy refractory colorectal cancer (*n* = 15) showed measurable levels of curcumin after doses between 0.45 and 3.6 g daily for up to 4 months. Curcumin was detected in plasma samples, after a dose of 3.6 g/day, at the 1 h time points on days 2, 8, and 29 of intervention with a mean of 11.1 ± 0.6 nmol/L. In these samples also, glucuronides (15.8 ± 0.9 nmol/L) and sulfates (8.9 ± 0.7) of curcumin were found. Moreover, patients under 3.6 g of curcumin daily displayed urinary levels between 0.1–1.3, 19–45, and 210–510 nmol/L of curcumin, curcumin sulfate, and glucuronides, respectively [28].

Patients with hepatic metastases from colorectal cancer (*n* = 12) received orally 0.45–3.6 g of curcumin daily, for one week before surgery. Trace levels (<0.01 µM) of curcumin and its conjugates (sulfate and glucuronide) were only found in liver and portal circulation [29].

Patients (*n* = 12) with confirmed colorectal carcinoma received 0.45, 1.8, or 3.6 g of curcumin daily, for seven days before colectomy displayed a poor concentration in the blood (<0.3 nmol/L). Levels of curcumin in normal and malignant colorectal tissue ranged from 0.9 to 20 nmol/g tissue depending on doses [30].

Another study enrolled healthy human volunteers to study curcumin pharmacokinetics after a single oral dose of 10–12 g. Free curcumin was detected in the plasma of only one subject after 30 min from the administration. The concentrations of curcumin glucuronide and curcumin sulfate at T_max_ at the 10 g dose level were 2.04 ± 0.31 µg/mL and 1.06 ± 0.40 µg/mL and at the 12 g dose level were 1.40 ± 0.74 µg/mL and 0.87 ± 0.44 µg/mL, respectively [31].

Another study in human volunteers (*n* = 4) was undertaken to investigate the pharmacokinetics of curcumin taken in turmeric-containing food: a sandwich made from turmeric-containing bread and a portion of soup, plus a sweet oat bar. Each subject consumed 3 g turmeric in total (approx. 100 mg of curcumin). Curcumin was investigated in plasma and detected in only 1/4 volunteers with a C_max_ of 3.2 nM at 2 h post-food ingestion. Significant metabolites of curcumin including curcumin glucuronide, demethoxycurcumin glucuronide, and curcumin sulfate were detected in all four volunteers with a C_max_ 47.6 ± 28.5 nM, C_max_ 1.9 ± 1.2 nM, and C_max_ 2.1 ± 1.7 nM respectively. These concentrations were achieved at 30 min post-food [32].

The viability of oral curcumin was also evaluated in gemcitabine-resistant patients with pancreatic cancer (*n* = 21) who received 8 g oral curcumin daily in combination with chemotherapy. Curcumin pharmacology was studied in *n* = 5 patients heterogeneous in terms of duration of curcumin treatment and time of blood draw after final curcumin intake. Total curcumin levels (comprehensive of phase II metabolites) ranged from 29 to 91 ng/mL, except for one patient who demonstrated a plasma curcumin level of 412 ng/mL (3 months of curcumin intake, curcumin titration was done 4 h after the final dose). This high plasma level was probably due to a decreased clearance of curcumin caused by constipation related to peritonitis carcinomatosa [33].

Another study on pancreas adenocarcinoma was done in which patients (*n* = 21) received daily 8 g of oral curcumin, with restaging every two months. In patients, (*n* = 19) curcumin pharmacokinetic profile was studied, and no free curcumin was detected in any of these. Conjugated curcumin was found to range between 0–125 ng/mL (2 h), 1.8–117.0 ng/mL (24 h), and 22–41 ng/mL (after 3 days) [34].

Chinese patients (*n* = 34) who present a decline in memory and cognitive function underwent six months of treatments with curcumin (1 g or 4 g daily). The maximum concentration in total curcuminoids was 92 ± 29 ng/mL [35].

The tolerability and efficacy in Alzheimer disease were also evaluated in patients (*n* = 36) who receive placebo, 2 grams/day, or 4 grams/day of oral curcumin for 24 weeks. The plasma levels of native curcumin and glucuronide were about seven ng/mL and 96 ± 26 ng/mL, respectively [36].

Levels of curcuminoids were quantified in colorectal mucosa of patients (*n* = 24) undergoing colorectal endoscopy or surgical resection. Fourteen days before endoscopic biopsy or colonic resection 2.35 g curcumin was administered. Curcuminoids were detectable in plasma samples (9/24), urine (24/24), and the colonic mucosa (23/24). Plasma levels of parent curcumin were 12.2 ng/mL and glucuronides 4.9 ng/mL. Tissue biopsies presented 48.4 mg/g (127.8 nmol/g) of curcumin which was demonstrated to persist in the mucosa for up to 40 h post-administration. In colorectal mucosa, samples were also detected curcumin metabolites and the other curcuminoids (demethoxycurcumin, bis-demethoxycurcumin).

The effects of oral curcumin (2 g or 4 g per day for 30 days) was assessed in 44 eligible smokers with eight or more aberrant crypt foci on screening colonoscopy. Concentrations of curcumin and curcumin conjugates were assayed in rectal mucosal biopsies and venous plasma. Tissue levels ranged from 3–11 µg/g protein and in blood 2.9–4.7 ng/mL. In plasma also curcumin conjugates were detectable ranging from 9.2–382.7 [37].

Curcumin, even though it displays poor oral bioavailability, due to its lipophilicity is able to cross the blood–brain barrier. Thus, is able to reach brain in biologically effective concentrations promoting neuroprotection. It should be noted that curcuminoid entry into the brain only if they are not glucuronidated. Very few studies, only in murine models, were realized to find its brain concentration. Mice administered 50 mg/kg curcumin by oral dose displayed a brain concentration inferior than limit of detection at 30, 60, 120 min after the administration. By contrast, with 100 mg/kg curcumin by intraperitoneal injection the concentration ranged between 4–5 µg/g tissue in a period between 20–40 min [38]. Mice chronically fed (4 months with 2.5–10 mg/day) with curcumin showed 0.5 µg/g tissue after oral administration, higher concentrations were reached with intraperitoneal and intramuscular administration [39].

Taken into consideration all these data, the administration of crude curcumin displayed a wide-ranging serum concentration from 1 to 3200 ng/mL depending on dose, ranging from 2 to 10 g, and subject’s physiology. In some cases, its concentration is under the instrumental limit of detection (<1 ng/mL) even with a dose of 3.6 g [30]. Thus, the choice of high-performance instrumentation, such as liquid chromatography coupled to mass spectrometry (LC-MS/MS), seemed to be fundamental for a correct curcumin pharmacokinetics determination.

## 3. Factors Impacting on Oral Curcumin Bioavailability

Polyphenols bioavailability in dietary sources can be affected by either origin, food processing, and macronutrients. Their levels and profile patterns depend, not only on the types of fruits and vegetables but also on climate, soil, plant stresses, ripening, and storage. Food processing such as grinding, drying and heating changes the food matrix and thus polyphenols composition. Moreover, macronutrients, especially dietary lipids, may affect curcumin solubility and absorption. Turmeric should be in cuisine associated with lecithin-rich ingredients like eggs or vegetable oils, in order to increase the dietary intake of curcuminoids (J. Cuomo et al., 2011). Moreover, powdered curcuminoids incorporated into buttermilk (300 mg curcuminoids/100 g of buttermilk, 0.3%), before yogurt manufacturing, resulted in increased in bioaccessibility (15-folds) of curcuminoids compared to that of neat curcuminoids. However, the enhanced bioaccessibility of curcuminoids in yogurt was still low (approximately 6%) (Fu et al., 2016). Digestive steps also contribute to low bioavailability of polyphenols affecting solubility, degradation in the intestinal environment, and the permeation rate in the small intestine.

There is evidence to suggest that gender can have a significant influence on curcumin pharmacokinetics. These differences may be due to gender-specific factors such as (1) increased clearance in men due to a higher activity of hepatic drug efflux transporters and (2) higher body fat in women [32,40]. Plasma levels, following administration of different oral curcumin formulation, to healthy human volunteers were 1.4 to 2.1 higher in women vs. men, depending on formulation [40]. By contrast, in another study which involved only one woman, plasma levels of the curcumin metabolites were all lower than in the male (*n* = 3) study participants (e.g., curcumin glucuronide 53.7 vs. 26.3 nM, curcumin sulfate 2.6 vs. 0.7 nM in male vs. female respectively) [32].

## 4. Enhancing Bioavailability of Oral Curcumin

In order to counteract poor curcumin absorption and rapid elimination from the body several strategies have been attempted such as the inhibition of curcumin metabolism with adjuvants as well as novel solid and liquid oral delivery systems. These novel drug delivery systems (Table 1) could overcome the pharmaceutical issue related to curcumin delivery enhancing the solubility, extending the residence in plasma, improving the pharmacokinetic profile and the cellular uptake [41,42].

### 4.1. Combination of Curcumin and Piperine

A natural product capable of modifying curcumin disposition and bioavailability is piperine, the natural alkaloid of black pepper (*Piper nigrum*), which is a potent inhibitor of biotransformation and especially glucuronidation. The association of 2 g of curcumin + 5 mg of piperine displayed a 3-fold increase [14], with respect to pure curcumin. Pharmacokinetics of curcumin+piperine (4 g + 24 mg) was studied in three groups (*n* = 8 each) of healthy volunteers under midazolam, flurbiprofen, and paracetamol. Concentrations of curcuminoids and piperine after administration of capsules were measurable, only after enzymatic hydrolysis, with a concentration, ranged from 136–176 ng/mL and did not differ between the different trials with paracetamol, flurbiprofen, or midazolam [43].

Healthy male volunteers (*n* = 8) after 2 g/kg of oral pure curcumin presented very low serum levels of curcumin (C_max_ 0.006 ± 0.005 µg/mL at 1 h) but when 2 g/kg of pure curcumin are combined with 20 mg/kg of piperine the concentrations are significantly increased (0.18 ± 0.16 µg/mL at 0.75 h) [44].

### 4.2. Association of Curcumin and Lecithin

Phospholipid-phytochemical complexes are purported to improve gastrointestinal absorption of poorly water-soluble phytochemicals via the amphipathic properties of the phospholipid [56].

A crossover study was carried out in healthy volunteers (*n* = 9) to measure plasma concentrations of curcuminoids after supplementation with two dosages of formulated curcuminoids mixture with lecithin (200 or 400 mg/day) and one dosage of non-formulated curcuminoid mixture (about 2 g/day). No plasma peak of free curcumin was detected in any plasma samples. After enzymatic hydrolysis, the concentrations of total curcuminoid were: (1) 206.9 ± 164.7 ng/mL at 2.7 ± 1 h after the administration of 400 mg of formulated preparation, (2) 68.9 ± 50.8 ng/mL at 3.3 ± 1 h after the administration of 200 mg of formulated preparation, and (3) 14.4 ± 12.5 ng/mL at 6.9 ± 6.7 h after the administration of non-formulated curcuminoid mixture. In respect of only curcumin, the concentrations (ng/mL) were 50.3 ± 12.7 at 3.8 ± 0.6 h (400 mg of formulated preparation), 24.2 ± 5.9 at 4.2 ± 0.8 h (200 mg of formulated preparation), and 9.0 ± 2.8 at 6.9 ± 2.2 h (crude curcumin powder) respectively. The phospholipids formulation with lecithin increased the bioavailability of curcuminoids [45].

In rats, peak plasma concentration and AUC were 5-fold higher for Meriva (a combination of curcumin-phospholipids) than for unbound curcumin [57]. Another small single-dose study demonstrated a comparable absorption of curcumin from 450 mg of Meriva and 4 g unbound of *Curcuma longa* [58].

In a study, plasma and rectal tissue concentrations of curcuminoid were studied between standard curcumin and phosphatidylcholine-complexed curcumin preparations. Plasma concentrations of curcumin and the major curcuminoids in curcumin extracts were similar even though the doses were 4 g for pure curcumin and 400 mg for phosphatidylcholine-complexed curcumin [46].

### 4.3. Curcumin in Hydrophilic Nanoparticles

Colloidal nanoparticles show a 15-fold increase in the concentration of administered curcumin as the result of an enhanced gastrointestinal absorption as a result of colloidal dispersion (Sasaki et al., 2011).

A water-soluble curcumin formulation containing turmeric extract, a hydrophilic carrier (polyvinyl pyrrolidone), cellulosic derivatives, and natural antioxidants (tocopherol and ascorbyl palmitate) was compared to standard curcumin in healthy volunteers. This novel formulation with an increased solubility provided a 46-fold increase in oral absorption as compared with the unformulated curcumin [48].

Theracurmin is a highly absorptive form of curcumin produced using a technique of a micro-particle and surface-controlled colloidal dispersion. A drinkable preparation of Theracurmin was studied, and a clinical study in healthy humans (*n* = 24) was undertaken in order to compare the reached plasma levels of curcumin after its ingestion vs. three other curcumin beverages sold in Japan. The blood AUC (0–8 h) was found to be 1.5- to 4.0-fold higher and, in the same way, its C_max_ was 1.8 to 3.8 times higher with Theracurmin than with the other three kinds of curcumin. These findings demonstrate that Theracurmin beverage shows the highest bioavailability among currently available beverages of curcumin [49].

Repetitive Theracurmin was tested in pancreatic or biliary tract cancer patients in order to evaluate its safety. Median plasma curcumin levels two h after Theracurmin administration were: 324 ng/mL with a dose of 200 mg of curcumin and 440 ng/mL with a dose of 400 mg of curcumin. These results were also combined with the evidence that the incidence of adverse events in cancer patients receiving gemcitabine-based chemotherapy was not increased by this curcumin treatment [50]. The same group tested this formulation also on healthy subjects (*n* = 9) with comparable results in terms of plasma concentration of curcumin: 189 ± 48 ng/mL with a dose of 150 mg and 275 ± 67 ng/mL with a dose of 210 mg [51].

### 4.4. Curcumin in the Lipid-Based Formulation

Nanostructured lipid carriers stand tall over other strategies, due to their remarkable features: (1) high bioavailability, (2) large loading capacity, (3) physical and chemical preservation, (4) controlled release, (5) the absence of chemical cross-linking, (6) low toxicity, and (7) well tolerated in multiple-dosage therapy.

Curcumin plasma concentration relationship was studied in both human volunteers and late-stage osteosarcoma patients. The formulation employed was 650 mg of either solid lipid particles (130–195 mg of curcumin) or >390 mg of curcumin extract. No plasma peaks were found in the group which underwent curcumin administration whereas with lipid vehicle curcumin concentration was proven to be 22.43 ng/mL at 2.4 h. The same lipid formulation was administered in osteosarcoma patients with a dose ranging from 2000 to 4000 mg reporting a maximum concentration between 30–40 ng/mL at 2–4 h. The use of this kind of formulation suggested a continuous absorption into the bloodstream through the colon [52].

Other studies on curcumin administration by lipids mixture or in complexation with phospholipids indicated only a mild increase in the bioavailability [57,59,60].

Liposomes are molecular assemblies like micro or nanospheres where lipids are organized in one or more bilayers surrounding an aqueous environment. They can be loaded with both hydrophilic and hydrophobic molecule, and to improve their stability they can be coated with polymers. In this, in vitro study, the bioavailability of curcumin was investigated in chitosan-coated liposomes containing curcumin as well as in loaded anionic liposomes. These two loading systems provided the same percentage of curcumin for absorption (bioaccessibility), but chitosan-coated liposomes delivered a higher concentration of bioactive curcumin (transformation). The results were a higher amount of curcumin in the bile salt micelles for curcumin loaded into chitosan-coated liposomes which displayed, indeed, a better bioavailability [15].

The potential application of sophorolipids, glycolipids composed of a polar sophorose group and a nonpolar fatty acid group, as biosurfactants for forming curcumin-loaded colloidal delivery systems was evaluated. The nanoparticles displayed a relatively high encapsulation efficiency (82%) and loading capacity (14%) for curcumin. In vivo bioavailability of curcumin was investigated by oral administration (100 mg/kg as pure curcumin or as curcumin micelles) to 12 male Sprague−Dawley rats. Curcumin oral administration with these nanoparticles led to a significantly higher (6-fold) and more prolonged level of curcumin in the blood of the rats than in the form of free crystals. These results were related to the ability of sophorolipid micelles to increase the solubilization of the curcumin in the mixed phase [61].

### 4.5. Curcumin in a Micellar System

Curcumin absorption and excretion kinetics were investigated in a single-blind crossover study with healthy female and male subjects comparing 500 mg formulation of native curcumin, curcumin incorporated into a micronised powder, or in liquid micelles. Micronized curcumin and micelles showed a significantly higher concentration of curcumin than curcumin powder both in plasma and in urine. Curcumin C_max_ in plasma was 7.1 nmol/L at 4 h, 41.6 nmol/L at 1.4, 3228 nmol/L at 1.1; whereas, in urine were 5.1 nmol/g creatine, 70.6 nmol/g creatine, and 753.4 nmol/g creatine in native curcumin, micronised powder, and liquid micelles respectively. In the same way, also demethoxycurcumin and bis-demethoxycurcumin were quantified [40].

Glioblastoma patients (*n* = 13) ingested 70 mg of micellar curcuminoids three times per day for four days (total amount of 689 mg curcumin, 134 mg demethoxycurcumin, and 17 mg bis-demethoxycurcumin) before brain surgery. Tumor and blood samples were taken during the surgery and analyzed for total curcuminoid concentrations: 56 pg/mg in resected tumors (range 9–151), and 253 ng/mL (range 129–364) in the blood. However, the intra-tumoral concentration might not be sufficient to cause significant localise antitumor effects [53].

### 4.6. Curcumin in Chitosan Nanoparticles

An in vivo pharmacokinetics study was done in albino rats to understand the differences between curcumin release by simple suspension, chitosan microspheres and ascorbic acid coated chitosan microspheres. The oral bioavailability of curcumin was enhanced to a great extent with microspheres containing ascorbic acid compared to pure curcumin and plain microspheres: 1.139 ± 0.118 vs. 0.512 ± 0.020 and 0.655 ± 0.028 μg/mL respectively. The presence of this acid might protect the drug from pH-mediated chemical degradation, thus improving the fraction of curcumin that can be absorbed. In that way, unmodified curcumin can reach the colon, after the dissolution of the delivery system and the degradation of chitosan by microbial flora [62].

Amorphous ternary nano complex of curcumin-chitosan-hypromellose exhibited superior (1) physical stability after 12-month storage, (2) dissolution characteristics, (3) solubility enhancement in simulated gastrointestinal fluids, and (4) minimal cytotoxicity towards human gastric epithelial cells. This nanocarrier may be promising to enhance curcumin solubility and thus its bioavailability [63].

Another system developed was a polyelectrolyte complex nanoparticles, with chitosan and acylated cruciferin. They demonstrated an encapsulation efficiency of 72% and a controlled in vitro release for 6 h of curcumin, using simulated gastro and intestinal fluids [64].

### 4.7. Miscellaneous

Healthy human subjects (*n* = 15) were administered with 500 mg Cureit, a bioavailable form of curcumin prepared by using polar-nonpolar-sandwich technology starting from complete natural turmeric matrix. The pharmacokinetic study results are also supported that Cureit can enhance the absorption of curcumin by the registration of high AUC and C_max_ values (527.1 ng h/mL, 74.3 ng/mL). Furthermore, this study simultaneously characterized and quantitatively in human plasma determined the metabolites of curcumin such as tetrahydrocurcumin, hexahydrocurcumin, octahydrocurcumin, curcumin glucuronide, curcumin sulfate along with the other curcuminoids [54].

The characteristics and bioavailability of ethyl cellulose–citric acid microspheres loading curcumin were studied in healthy rats (*n* = 40). The drug concentration was 4-fold higher than pure curcumin with a maximum peak value (42.51 ng/mL) at 8 h. Moreover, the peak time was delayed for 7.5 h [65].

BCM-95CG (Biocurcumax™) is a simple and cost-effective formulation which consisted of a mixture of sesquiterpenoids present in turmeric (less than 5% in essential oils especially Ar-turmerone) and the curcuminoids providing an enhanced bioavailability. Healthy subjects under the age group of 28–50 (*n* = 11) were recruited for the study and divided into three separate groups: (1) group 1 (*n* = 4) consumed 4 × 500 mg capsules of Biocurcumax™; (2) group 2 (*n* = 4) consumed 2000 mg control curcumin; and (3) group 3 (*n* = 3) consumed 2000 mg curcumin-lecithin-piperine formula. The proprietary formulation Biocurcumax™ largely enhance the bioavailability of curcumin. The mean plasma concentration of curcumin from Biocurcumax™ was 689.18 ng/g at 4.67 h whereas from curcumin-lecithin-piperine formula reached a peak value of 344.32 ng/g at 3.5 h and control curcumin 149.8 ng/g at two h. The main differences found in the proprietary formulation vs. curcumin were (1) higher concentrations which gradually decreased even at 8 h some residual curcumin remained in the blood; (2) a possible extra-intestine absorption; and (3) due to its long presence in circulation may also modulate the activity of P-glycoprotein and the Phase II enzymes. Results from that pilot study indicate that curcumin is absorbed early and retained longer from the BCM-95CG (Biocurcumax™) showing a better pharmacokinetic profile compared both to pure curcumin and curcumin-lecithin-piperine formula [55].

Curcumin solubility can be enhanced through whey protein-based encapsulation giving a higher bioavailability and bio-efficacy by enhancing curcumin solubility with a slow release: about 50% at 24 h and 70% at 48 h (in vitro evidence) [66].

## 5. Conclusions

Curcumin has been used for thousands of years in Asian traditional medicine for its anti-inflammatory and anti-oxidative profile. Although oral curcumin supplementation has shown therapeutic efficacy in many clinical trials, its nutraceutical activities are counteracted by low absorption, restricted bioavailability, fast metabolism, and elimination. Some pharmaceutical technology or peculiar combination with other compounds, such as piperine or lecithin, were found to enhance its solubility, extend the residence in plasma, and improve the pharmacokinetic profile and the cellular uptake. Depending on the dose and the pathophysiological condition of the subject, the administration of crude curcumin showed a wide range in serum concentrations (1–3200 ng/mL) and in some cases, the concentration is even not determinable. Different novel delivery systems such as solid lipid particle, micellar system, or hydrophilic nanoparticles could increase curcumin concentration up to 15–20 fold. The determination of curcumin pharmacokinetic profile seems to be challenging, in many cases, due to low circulant concentrations. Thus, the choice of an advanced analytical technique, such as LC-MS/MS, should be considered.

To conclude, the health potential of curcumin, that is undoubtedly assessed, has to be always evaluated by correlation with a well-defined way of administration, in order to achieve the optimal efficacy.

## Figures and Tables

**Table 1:** Enhancing bioavailability of oral curcumin by different novel delivery systems.

Formulation	Subjects ^1^	Curcumin Dose	Pharmacokinetics Parameters (Curcumin) ^2^	Ref.
Curcumin + piperine	H	2 g + 5 mg	6.92 ng/mL (mean)	[14]
	H	4 g + 24 mg	136–176 ng/mL (range)	[43]
	H	2 g/kg + 20 mg/kg	180 ng/mL at 0.75 h	[44]
Curcumin + lecithin	H	400 mg	50.3 ± 12.7 ng/mL at 3.8 ± 0.6 h	[45]
	H	200 mg	24.2 ± 5.9 ng/mL at 4.2 ± 0.8 h	[45]
	H	400 mg	71 ng/mL (mean)	[46]
Curcumin in hydrophilic nanoparticles	H	30 mg	1.8 ± 2.8 ng/mL	[47]
	H	376 mg	27.3 ± 6.4 ng/mL at 1.4 h	[48]
	H	30 mg	25.5 ± 12.2 ng/mL	[49]
	P	Multiple doses of 200 or 400 mg/day	324 ng/mL with a dose of 200 mg of Theracurmin and 440 ng/mL with a dose of 400 mg	[50]
	H	150 or 210 mg	189 ± 48 ng/mL with a dose of 150 mg and 275 ± 7 ng/mL with a dose of 210 mg	[51]
Curcumin in solid lipid particle	H	650 mg (135–195 mg curcumin)	22.4 ng/mL at 2.4 h	[52]
	P	From 2 to 4 g	30–40 ng/mL between 2 to 4 h	[52]
Curcumin in micellar system	H	500 mg	1189 ng/mL at 1.1 h	[40]
	P	210 mg/day per 4 days	253 ng/mL (total curcuminoids)	[53]
Miscellaneous	H	500 mg Cureit	74.3 ng/mL	[54]
	H	4 × 500 mg capsules of Biocurcumax™	689.18 ng/g at 4.6 h	[55]

^1^ H: healthy volunteers P: patients. ^2^ In here is reported the maximum concentration of curcumin in systemic blood otherwise when the data was not shown, the range or the mean of curcumin concentration were implemented.

## Abbreviations

AUC	area under the curve or integral of the plasma drug concentration-time curve
SULTs	sulfotransferase
UGTs	uridine 5’-diphospho-glucuronosyltransferase
NADPH	nicotinamide adenine dinucleotide phosphate
PGP	P-glycoprotein
C_max_	maximum serum concentration
T_max_	time to reach maximum concentration
LC-MS/MS	liquid chromatography coupled to mass spectrometry
